# Rates of colorectal surgery in patients with non-malignant colorectal polyps: Results from a nationwide study

**DOI:** 10.1055/a-2795-7563

**Published:** 2026-02-11

**Authors:** Saqr Alsakarneh, Rahul Karna, Aasma Shaukat, Mohammad Bilal

**Affiliations:** 1 6915 Gastroenterology & Hepatology, Mayo Clinic, Rochester, United States; 2 311816 Gastroenterology & Hepatology, University of Minnesota System, Minneapolis, United States; 3 12297 Gastroenterology & Hepatology, NYU Langone Health, New York, United States; 4 129263 Gastroenterology & Hepatology, University of Colorado Anschutz Medical Campus, Aurora, United States

**Keywords:** Endoscopy Lower GI Tract, Colorectal cancer, Polyps / adenomas / ..., Epidemiology

## Abstract

Despite advances in endoscopic techniques, many colorectal surgeries in the United States are still performed for non-malignant colorectal polyps (NMCRPs). This study evaluated trends, demographic variations, and outcomes of surgeries for NMCRPs among all colorectal surgeries over the past decade. Using the TriNetX nationwide database, we identified adults (≥ 18 years of age) who underwent colectomy or proctectomy for NMCRPs or colorectal cancer between 2013 and 2023. We evaluated the proportion of surgeries performed for NMCRPs, stratified by demographic factors, and compared postoperative adverse events (AEs) between NMCRP and colorectal cancer surgeries. Among 136,721 surgeries, 52,480 (38.4%) were for NMCRPs. The proportion of NMCRP surgeries decreased from 59% in 2013 to 33% in 2023, with the most significant decline between 2013 and 2016. Black individuals showed the highest decrease. Compared with colorectal cancer surgeries, NMCRP surgeries were associated with significantly lower risks of wound, infectious, urinary, pulmonary, gastrointestinal, and cardiac AEs. Although the proportion of NMCRP surgeries has declined, ongoing efforts in education and training are needed to further reduce unnecessary surgeries and improve patient outcomes.

## Introduction

Despite advances in endoscopic resection techniques, recent reports indicate that a significant number of colorectal surgeries in the United States are still being performed for non-malignant colorectal polyps (NMCRPs) 1 . Advanced endoscopic resection techniques, which are both effective and safe, can be used to manage the majority of large NMCRPs 2 . Current guidelines recommend referral to an endoscopist with expertise in managing large NMCRPs for repeat colonoscopy to attempt endoscopic resection, before opting for surgical resection 3 . In this study, we aimed to evaluate trends, demographic variations, and outcomes of surgical interventions for NMCRPs over the past decade using a nationwide database.

## Patients and methods

### Cohort definition

We queried clinical data from the US-Collaborative network in TriNetX, a large multi-institutional nationwide database, which aggregates healthcare data from 64 healthcare organizations in the United States comprising over 105 million patients 4 . The TriNetX platform has previously been used and validated for retrospective cohort studies in the US 5 . TriNetX uses International Classification of Diseases (ICD)-10 codes as well as Current Procedural Terminology (CPT) codes to identify diagnoses and procedures. All adult patients (≥ 18 years old) patients who underwent elective colectomy or proctectomy and had a diagnosis of either NMCRP or colorectal cancer (CRC) were identified. To exclude any polyps that might be incidental findings, we excluded patients with intestinal perforation, inflammatory bowel disease, diverticulitis, and all patients who underwent total colectomy 1 .

The patient sample was divided into two cohorts. The first cohort was composed of patients who had medical encounters for NMCRPs. The second cohort was composed of patients with CRC. For each calendar year, the denominator was the total number of colorectal surgeries (for NMCRPs and CRC), and the numerator was the number of surgeries for NMCRPs.

### Statistical analysis

We used the TriNetX built-in analytics platform for all statistical analyses. Continuous variables were presented as means with standard deviations (SD) and compared using independent samples *t* -tests. Categorical variables were presented as counts and percentages and compared using chi-square tests. To adjust for baseline differences between patients with NMCRPs and CRC, we performed 1:1 propensity-score matching using logistic regression. Matching variables included demographics (age, sex, race, ethnicity) and comorbidities. A greedy nearest-neighbor matching algorithm with a caliper of 0.1 pooled SD was applied without replacement. After matching, standardized mean differences were calculated to assess covariate balance. Odds ratios (ORs) with 95% confidence intervals (CIs) were reported for postoperative complications. Time-trend analyses were performed using Joinpoint regression to calculate average annual percentage change (AAPC) with statistical significance assessed at *P* < 0.05.

### Aims

The primary aim was to examine the proportion of surgeries performed for NMCRPs among all colorectal surgeries, stratified by age, sex, race, and ethnicity. Secondary aims included: 1) temporal trends in NMCRP surgery proportions; and 2) comparison of postoperative adverse events (AEs) between NMCRP and CRC surgeries.

## Results

Of 136,721 colorectal surgeries from 2013 to 2023, 52,480 (38.4%) were for NMCRPs. Among 52,480 patients with NMCRPs, the mean (SD) age was 64.1 years (12.4) and 45.6% were females. Among 84,241 patients with CRC surgeries, the mean (SD) age was 64.4 years (12.7), with 45.6% females. Table 1 shows the comparison of demographic details between study cohorts.

**Table:** **Table 1** Baseline demographic and comorbidity characteristics for patients undergoing surgery for non-malignant colorectal polyps versus colorectal cancer, both before and after propensity-score matching.

	Before propensity score matching			After propensity score matching		
	Non-malignant polyps	Colorectal cancer	*P* value	Non-malignant polyps	Colorectal cancer	*P* value
Demographics						
Age, years, mean (standard deviation)	64.1 (12.4)	64.7 (13.3)	< 0.001	64.1 (12.4)	64.4 (12.7)	0.001
Gender, female, n (%)	23,931 (45.6%)	38,699 (45.9%)	0.222	21,510 (45.80%)	21,410 (45.60%)	0.513
Race/ethnicity, n (%)						
White	34,581 (65.9%)	57,127 (67.8%)	< 0.001	31,315 (66.60%)	32,002 (68.10%)	< 0.001
African American	7,902 (15.1%)	9,447 (11.2%)	< 0.001	6,514 (13.90%)	6,093 (13.00%)	< 0.001
Hispanic	2,475 (4.7%)	4,959 (5.9%)	< 0.001	2,310 (4.90%)	2,096 (4.50%)	0.001
Asian	1,969 (3.8%)	4,168 (4.9%)	< 0.001	1,878 (4.00%)	1,769 (3.80%)	0.066
Comorbidities, n (%)						
Essential (primary) hypertension	32,809 (62.5%)	36,728 (43.6%)	< 0.001	27,608 (58.70%)	28,668 (61.00%)	< 0.001
Diabetes mellitus	16,777 (32.0%)	16,788 (19.9%)	< 0.001	13,202 (28.10%)	13,338 (28.40%)	0.324
Unspecified dementia	1,284 (2.4%)	1,322 (1.6%)	< 0.001	1,013 (2.20%)	986 (2.10%)	0.542
Human Immunodeficiency Virus	354 (0.7%)	397 (0.5%)	< 0.001	275 (0.60%)	261 (0.60%)	0.544
Heart failure	9,922 (18.9%)	7,438 (8.8%)	< 0.001	6,749 (14.40%)	6,422 (13.70%)	0.002
Ischemic heart diseases	15,212 (29.0%)	14,261 (16.9%)	< 0.001	11,544 (24.60%)	11,383 (24.20%)	0.221
Cerebrovascular diseases	7,842 (14.9%)	6,718 (8.0%)	< 0.001	5,725 (12.20%)	5,595 (11.90%)	0.193
Chronic lower respiratory diseases	15,242 (29.0%)	13,581 (16.1%)	< 0.001	11,436 (24.30%)	11,431 (24.30%)	0.97
Peptic ulcer disease	1,181 (2.3%)	713 (0.8%)	< 0.001	720 (1.50%)	650 (1.40%)	0.057
Alcoholic liver disease	1,725 (3.3%)	647 (0.8%)	< 0.001	1,278 (2.70%)	524 (1.10%)	< 0.001
Nicotine dependence	11,776 (22.4%)	10,030 (11.9%)	< 0.001	8,652 (18.40%)	8,594 (18.30%)	0.625
Overweight and obesity	14,077 (26.8%)	13,427 (15.9%)	< 0.001	10,981 (23.40%)	11,077 (23.60%)	0.46
Chronic kidney disease	10,456 (19.9%)	8,280 (9.8%)	< 0.001	7,429 (15.80%)	7,151 (15.20%)	0.012
Liver diseases	9,616 (18.3%)	11,772 (14.0%)	< 0.001	7,754 (16.50%)	7,233 (15.40%)	< 0.001
Alcohol abuse	3,547 (6.8%)	2,256 (2.7%)	< 0.001	2,261 (4.80%)	2,107 (4.50%)	0.017
Malnutrition	4,036 (7.7%)	5,248 (6.2%)	< 0.001	3,310 (7.00%)	2,989 (6.40%)	< 0.001

**Fig. 1:**
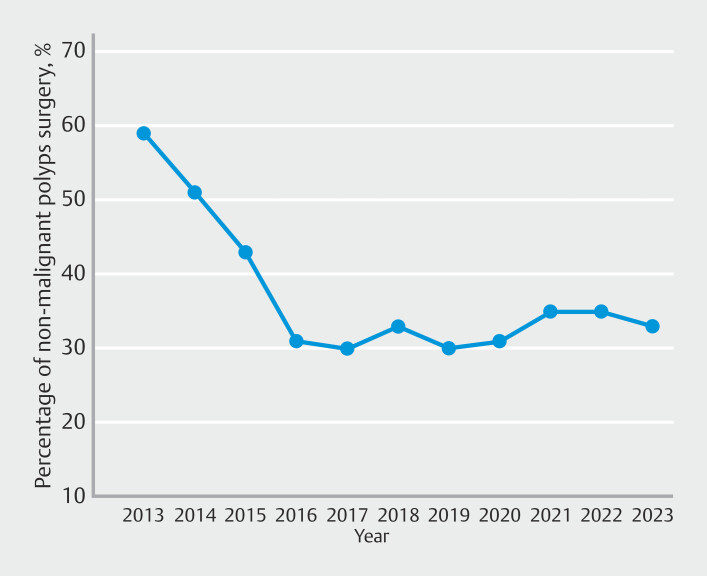
Overall time-trend analysis of rates of surgeries for non-malignant colorectal polyps.

**Fig. 2:**
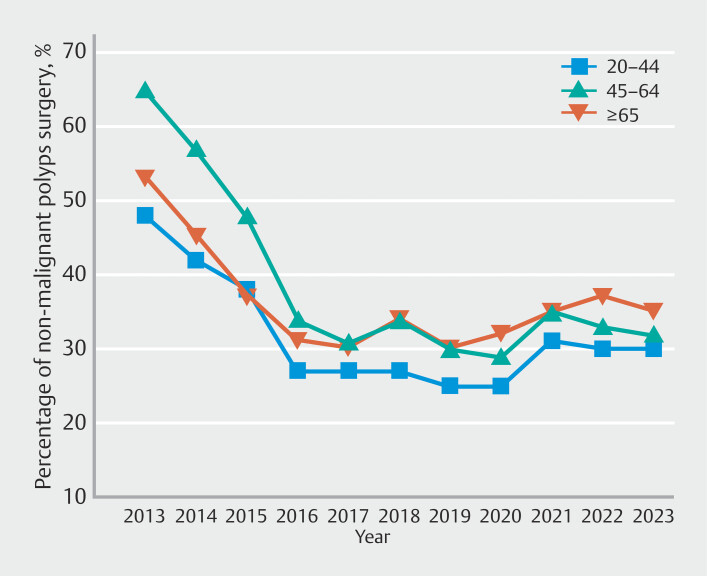
Age-specific time-trend analysis of rates of surgeries for non-malignant colorectal polyps.

**Fig. 3:**
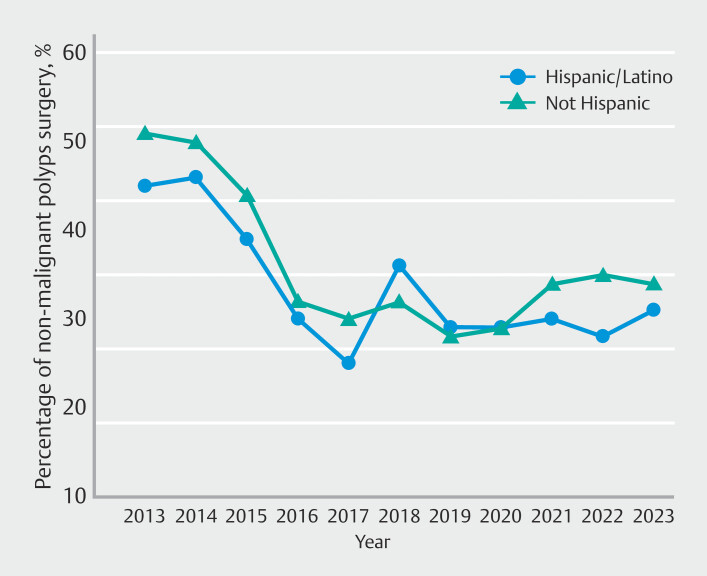
Ethnicity-specific time-trend analysis of rates of surgeries for non-malignant colorectal polyps.

**Fig. 4:**
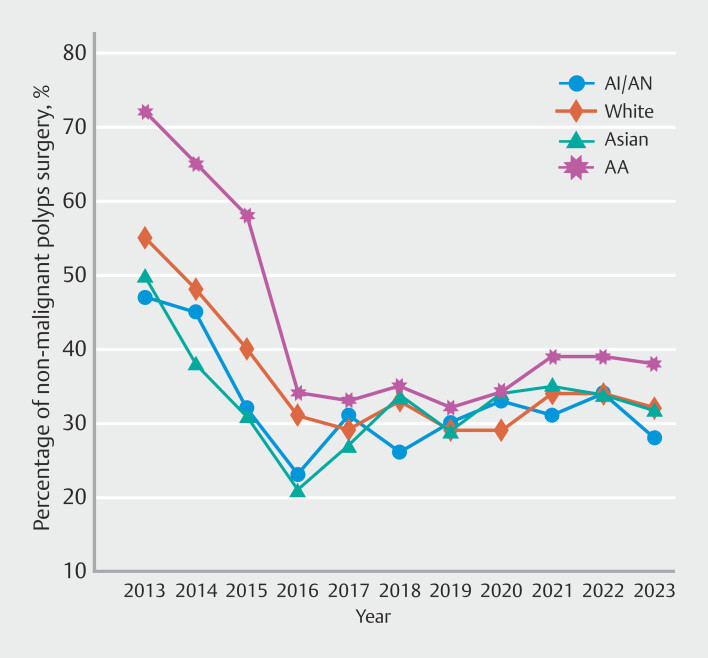
Race-specific time-trend analysis of rates of surgeries for non-malignant colorectal polyps.

**Fig. 5:**
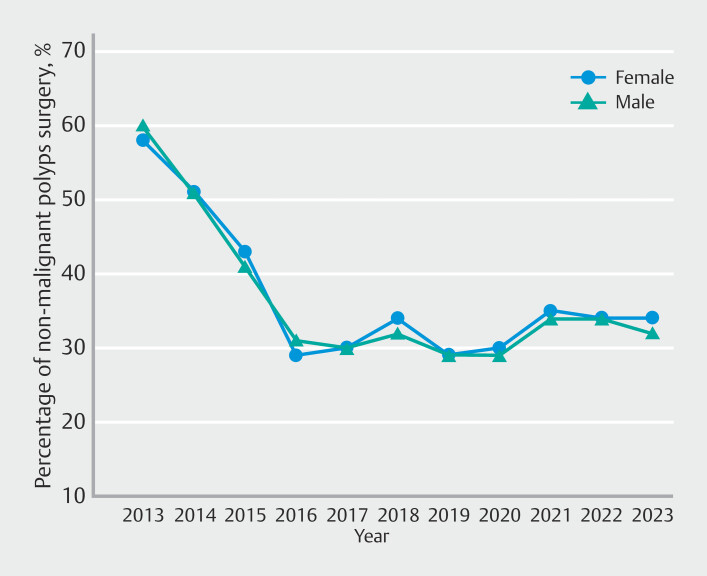
Gender-specific time-trend analysis of rates of surgeries for non-malignant colorectal polyps.

From 2013 to 2023, there was a decreasing trend of proportions of surgery for NMCRPs from 59% to 33%, with an AAPC of -5.9% (95% CI -6.5% to -5.3%). The largest decrease was during 2013 to 2016 (AAPC -20.5%; 95% CI -21.5% to -19.5%) as shown in Fig. 1 . For age, although all age groups experienced decreasing trends, the largest decrease was in patients aged 45 to 64 years (AAPC -6.9%; 95% CI -7.6% to -5.4%) ( Fig. 2 ). There was no difference between Hispanics and non-Hispanics (AAPC -4.5 vs -4.6%, respectively) with no statistically significant difference ( Fig. 3 ). In the temporal trend analysis, rates declined over time within each racial group. Black individuals experienced the greatest decline over time (AAPC −6.6%; 95% CI −7.5% to −6.1%), followed by White individuals (AAPC −5.5%; 95% CI −6.2% to −4.8%) ( Fig. 4 ). In the trend analysis, rates declined over time for both males and females (AAPC −6.4% for males and −5.9% for females), reflecting decreases from earlier to later time periods for each sex ( Fig. 5 ). When analyzed by type of surgery, 12,545 patients (24%) underwent proctectomy. There was no significant difference in the trends observed for the decline in both colon and rectal surgeries.

Among patients who underwent surgical resection for NMCRPs, 76% underwent colectomy whereas 24% underwent proctectomy, 8% experienced postoperative AEs, with infectious (2.55%) and cardiac (2.25%) AEs being the most common. When compared directly with CRC surgeries, NMCRP surgeries were associated with significantly fewer postoperative AEs across multiple domains ( Table 2 ). NMCRP surgeries were associated with decreased risk of postoperative wound (OR 0.39), infections (OR 0.48) and gastrointestinal AEs (OR 0.36) compared with CRC surgeries.

**Table:** **Table 2** Summary of postoperative adverse events comparing non-malignant colorectal polyps versus colorectal cancer surgeries, using logistic regression adjusted for matched cohorts.

	Non-malignant polyps, n (%)	Colorectal cancer, n (%)	Adjusted odds ratio (95% CI)	*P* value
Postoperative wound adverse events	105 (0.22)	264 (0.56)	0.396 (0.316–0.497)	< 0.0001
Postoperative infectious adverse events	1,212 (2.55)	2,448 (5.15)	0.482 (0.449–0.517)	< 0.0001
Postoperative urinary adverse events	35 (0.07)	102 (0.22)	0.343 (0.233–0.503)	< 0.0001
Postoperative pulmonary adverse events	275 (0.58)	475 (0.99)	0.576 (0.497–0.669)	< 0.0001
Postoperative gastrointestinal adverse events	781 (1.64)	2,125 (4.47)	0.357 (0.329–0.388)	< 0.0001
Postoperative cardiac adverse events	1,068 (2.25)	1,398 (2.94)	0.759 (0.7–0.822)	< 0.0001
Intraoperative and other adverse events	350 (0.74)	454 (0.96)	0.769 (0.669–0.885)	< 0.0001
CI, confidence interval.				

## Discussion

Our analysis revealed a significant decline in need for surgery for NMCRPs, decreasing from 59% in 2013 to 33% in 2023. The most notable decline occurred between 2013 and 2016, with an AAPC of -20.5%.

When examining demographic trends, we observed that all age groups experienced a decline in NMCRP surgeries, with the most substantial reduction in individuals aged 45 to 64 years, suggesting this age group may have benefitted the most from early adoption of endoscopic techniques. In addition, racial variations were evident, with Black individuals experiencing the greatest decrease. These findings suggest that educational and procedural advancements have been effective across diverse populations, although the reason behind these racial differences is yet to be understood.

Although the significant decrease in rate of surgery for NMCRPs is encouraging, over one-third of colorectal surgeries in 2023 were still performed for NMCRPs. Previous reports have shown that approximately 90% of complex NMCRPs, regardless of size and location, can be safely resected endoscopically as an outpatient procedure 6 . A previous meta-analysis showed that 14% of patients with colorectal polyps were referred to surgery before any attempt at endoscopic resection 6 . Therefore, our analysis suggests the need for continued efforts to identify patient- and endoscopist-level factors contributing to these findings and robust educational interventions to reduce rates of surgery for NMCRPs. Possible factors for decreasing surgery rates for NMCRPs include availability and accessibility of endoscopists with expertise in advanced tissue resection, knowledge of advanced resection techniques in endoscopists detecting large polyps, and patient preference for minimally invasive procedures 1 . In addition to focusing interventions on endoscopists detecting and referring these NMCRPs to surgery, efforts should also be made to highlight advancement in endoscopic techniques to surgical colleagues.

Patients with CRC cancer had higher rates of postoperative AEs including wound, infectious, and gastrointestinal AEs compared with those with NMCRPs. However, rates of AEs with surgery for NMCRPs are not trivial with approximately 8% of patients experiencing postoperative AEs in this study. In comparison, polypectomy is associated with an AE rate of 0.42%, although endoscopic mucosal resection (EMR) can be associated with AEs in up to 1.3% 7 . Endoscopic submucosal dissection (ESD) utilized for resection of suspected superficially invasive CRC or lesions with submucosal fibrosis is associated with AE rates of 4.8% 7 . Endoscopic full thickness resection (EFTR) is utilized for removal of non-lifting polyps and can be associated with AEs in 9.9% of patients, with the majority improving with conservative management 7 . These endoscopic resection techniques are organ-preserving, can obviate the need for ostomy, and are associated with significantly improved quality of life (QoL0. Moreover, approximately one-fourth of surgeries for NMCRPs were performed in the rectum. This is an important consideration because rectal surgeries are typically associated with higher morbidity and impact on QoL as compared with surgeries in the colon 8 .

Despite, decreasing trends, a significant proportion of NMCRPs still involved surgery. Widespread dissemination, training of endoscopic resection techniques including EMR, ESD, and EFTR to trainees and general gastroenterologists is required to help diminish the need for surgery, especially in the West. The European Society of Gastrointestinal Endoscopy outlines a comprehensive curriculum including prerequisites in colonoscopy and basic polypectomy skills prior to EMR training 9 . The curriculum entails formal didactics, in-vivo training, and performance of supervised procedures with monitoring of key performance indicators, with emphasis on optical diagnosis, appropriate selection of techniques, and rigorous training in technique to improve complete resection rates, and thus, potentially decrease the need for surgery. Barriers exist to receive quality ESD training in the United States. There are a limited number of established ESD training programs, decreased availability of gastric ESD, which is often considered as the stepping stone prior to colorectal ESD, and lack of reimbursement for ESD, which is a technically complex and resource-intensive technique. However, recognition of these barriers offers an opportunity to improve quality of training in endoscopic resection techniques, which will eventually help reduce rates of surgery for non-malignant polyps. Implementation of multidisciplinary teams consisting of endoscopists and surgeons for lesion assessment, and resection technique selection could also help reduce surgery rates for non-malignant lesions. Incorporation of magnification chromoendoscopy and ESD as part of standard management of complex colorectal polyps can alleviate the number of surgeries for NMCRPs 10 . Recently, a CPT code for ESD has been approved in the United States, which will help alleviate one of the barriers to ESD training in the country.

Our study is not without its limitations. Being a database study, the analytic cohort is subject to coding errors. The cohort selection was based upon ICD and CPT codes, and individual patient-level data were not available to verify polyp histology. We could only assess proportions of surgery performed for NMCRPs over the years, rather than true incidence of surgery rates due to lack of data on total number of polyps in the included population.

Management of malignant polyps is still evolving in the West, and based on local expertise and institutional framework, hence, it was not assessed as part of our study. The decision to pursue surgery is based upon multiple factors including comorbidities, surgical fitness, and stage of disease. We could not perform analysis of the impact of preoperative comorbidities on proportions of surgery for NMCRPs compared with CRC.

## Conclusions

In conclusion, although the decreasing trend in surgery for NMCRPs is promising, ongoing efforts are necessary to further reduce surgery for these polyps. Emphasizing education, accessibility, and training on advanced endoscopic resection techniques will be critical to achieving this goal. Identification of factors leading to surgery for NMCRPs and interventions tailored to these factors are needed.
